# Luminescent Silicon Nanowires as Novel Sensor for Environmental Air Quality Control

**DOI:** 10.3390/s22228755

**Published:** 2022-11-12

**Authors:** Dario Morganti, Maria José Lo Faro, Antonio Alessio Leonardi, Barbara Fazio, Sabrina Conoci, Alessia Irrera

**Affiliations:** 1 Department of Chemical, Biological, Pharmaceutical and Environmental Sciences, University of Messina, Viale Ferdinando Stagno d’Alcontres 5, 98166 Messina, Italy; 2 Department of Physics and Astronomy, University of Catania, Via Santa Sofia 64, 95123 Catania, Italy; 3 CNR-IMM UoS Catania, Via Santa Sofia 64, 95123 Catania, Italy; 4 URT LAB SENS, Beyond Nano—CNR, c/o Department of Chemical, Biological, Pharmaceutical and Environmental Sciences, University of Messina, Viale Ferdinando Stagno d’Alcontres 5, 98166 Messina, Italy; 5 CNR-IMM Istituto per la Microelettronica e Microsistemi, Zona Industriale, VIII Strada 5, 95121 Catania, Italy

**Keywords:** silicon nanowires, gas sensing, photoluminescence, optical detection, electrical detection

## Abstract

Air quality monitoring is an increasingly debated topic nowadays. The increasing spillage of waste products released into the environment has contributed to the increase in air pollution. Consequently, the production of increasingly performing devices in air monitoring is increasingly in demand. In this scenario, the attention dedicated to workplace safety monitoring has led to the developing and improving of new sensors. Despite technological advancements, sensors based on nanostructured materials are difficult to introduce into the manufacturing flow due to the high costs of the processes and the approaches that are incompatible with the microelectronics industry. The synthesis of a low-cost ultra-thin silicon nanowires (Si NWs)-based sensor is here reported, which allows us the detection of various dangerous gases such as acetone, ethanol, and the ammonia test as a proof of concept in a nitrogen-based mixture. A modified metal-assisted chemical etching (MACE) approach enables to obtain ultra-thin Si NWs by a cost-effective, rapid and industrially compatible process that exhibit an intense light emission at room temperature. All these gases are common substances that we find not only in research or industrial laboratories, but also in our daily life and can pose a serious danger to health, even at small concentrations of a few ppm. The exploitation of the Si NWs optical and electrical properties for the detection of low concentrations of these gases through their photoluminescence and resistance changes will be shown in a nitrogen-based gas mixture. These sensing platforms give fast and reversible responses with both optical and electrical transductions. These high performances and the scalable synthesis of Si NWs could pave the way for market-competitive sensors for ambient air quality monitoring.

## 1. Introduction

In recent years, technological advancements and the emergence of ever more numerous industries have had a huge impact on the growing increase in the production of dangerous gases. VOCs, CO, CO_2_, NO_x_, NH_3_, and various organic compounds are a few examples of the many toxic gases released into the environment due to industrial manufacturing processes, automotive and even domestic instrumentation exhausts. For example, the health condition of an average human being can be seriously compromised by various hazardous gases in the environment, so they must be detected and removed mainly indoors [1,2,3,4]. The irreversible consequences of these dangerous gases on human health have made gas detection a priority request. Thus, the demand for scalable, low-power operating gas sensors has grown more and more over the years.

Currently, gas sensors assume a key role due to their wide applications in medicine, the environment, road safety and the automotive industry and smart cities [5,6,7,8,9,10]. Indeed, air quality monitoring is a critical aspect for various industrial sectors such as manufacturing industries, pharmaceutical industries, refineries, aircraft, shipbuilding, agriculture and wastewater treatment plants [11,12,13].

Among all the toxic compounds, acetone, ethanol and ammonia are certainly substances worthy of attention. Acetone is a very diffused Volatile Organic Compound (VOC). In particular, it is one of the most widespread organic compounds due to its use both in research and industrial processes, as a reactive intermediate as well as a solvent or surface cleaner. [14]. It is widely used in fields of application such as plastic manufacturing, drug synthesis, printing, optoelectronics, and microscopy [15]. When inhaled, acetone can be extremely dangerous, causing severe headaches, coughs, and in worst cases, can severely affect the central nervous system [16]. Eight-hour occupational exposure limits generally range from 750 ppm; however, the onset of the first symptoms as irritation of the upper respiratory airways or mucosal membranes is found at lower concentrations as 250 ppm for a 4 h exposure [17].

Another substance worthy of particular control is ethanol. Excessive consumption of ethanol can cause alcohol dependence, heart disease and can aggravate all types of injuries and trauma. Furthermore, it was documented that ethanol can lead to malnutrition and can manifest a toxicological effect due to its interference with hepatic metabolism and immunological functions [18]. In addition, a link between alcohol and various types of cancer has been documented [18]. Among all these effects, certainly the most recurrent consequences are linked to episodes of fights, violence and road accidents. Road safety regulations impose a limit of 50 ppm on the maximum detectable level of alcohol in a driver. This is a crucial aspect for the realization of new sensors that can be used both by the police forces and for self-monitoring of the alcohol level.

Ammonia is a toxic gas with a strong and pungent smell. It is present in the atmosphere as a product released both in industrial and natural processes [19]. Inhaling ammonia is very harmful to health, as it can cause very serious diseases such as permanent lung damage and kidney failure [20]. The threshold limit value for ammonia set by the Occupational Safety and Health Administration (OSHA) is 50 ppm averaged over an eight-hour workday. This is the standard that must be met in every workplace. Therefore, the detection of these hazardous gases has increasingly become a necessity in order to minimize their impact on human health and the environment.

In this scenario, building selective, sensitive, reliable and cost-effective gas detection platforms for these hazardous gases sensing is still a priority demand. The applications of nanostructured materials have become increasingly widespread with the development of recent miniaturization techniques. The particular physico-chemical properties of nanostructures are increasingly arousing the interest of the entire scientific community for numerous applications [21,22,23,24,25,26,27,28,29,30]. Consequently, different detection devices have been conceived for its use in different sectors, such as in chemistry, biology, the environment and biomedicine [31,32,33,34,35,36,37,38,39,40]. In recent years, designing novel sensors based on nanostructured materials has been a highly debated matter. In fact, their high surface/volume ratio (S/V) allows a stronger interaction with external molecules improving their detection behavior. Different types of nanomaterials have been used for the realization of sensors as new assets for different application fields [41,42,43,44]. However, their diffusion and technological transfer are commonly limited by the material cost and manufacturing processes, which are often incompatible with industrial fabrication lines.

The most commonly used sensing materials are inorganic metal oxide semiconductors. These types of sensors are mainly based on the variation of the resistance following the interaction with the target gas through redox reactions between the target and the surface of the metal oxide [45]. However, these processes often require very high working temperatures [1,46]. This is a drawback for low-cost industrial applications, especially nowadays when the reduction of energy consumption is increasingly sensitized.

The realization of sensors based on silicon nanostructures can match both the high performances of the nanostructured materials and the availability of a large-scale production, thus having an impressive effect on technological transfer and the market. In fact, silicon is the main element in our microelectronics technology, as it is widely available in nature, has low extraction costs, and is easy to prepare and integrate for the realization of different devices. Among the various types of silicon-based nanomaterials present in literature, silicon nanowires (Si NWs) benefit from a very high surface-to-volume ratio, simple and quick synthesis, tunable structural properties, and environmental compatibility. For all these reasons, they are emerging as excellent materials for building sensor devices [47,48,49,50,51].

In literature, Si NWs-based sensors mainly exploit the variation of their surface electrical conductivity as a transduction mechanism [52,53,54]. While the realization of a new detection platform based on Si NWs photoluminescence (PL) at room temperature (RT) was only recently demonstrated by our group. The performances of this sensor have been demonstrated for the detection of biomarkers as DNA and extracellular vesicle [55,56]. Silicon is an extremely poor light emitter as it is an indirect bandgap semiconductor. So, in order to exhibit room temperature luminescence, it is necessary to realize extremely small silicon nanostructures in order to have a quantum confinement effect. Although porous silicon [57,58] and silicon nanocrystals [59,60] are Si-based nanostructures in which light emission has been observed, they suffer from numerous limitations, such as light-emission instability and aging effect [61,62], which severely hamper their sensing applications.

Many Si NWs-based sensors for the detection of different gaseous substances have already been realized in order to match the current gas sensing demand. However, for most of them, the surface functionalization or decoration with metal nanoparticles is carried out aiming to improve their electrical performances [63,64,65].

As an alternative to gas detection devices based on electrical performance, optical sensors emerge which are not affected by electrical noise drawbacks, and for this reason are considered more reliable [66,67]. Nevertheless, in literature, a chemical sensor based on the RT luminescence of Si NWs was absent up to a few years ago. This was due to the traditional techniques used for the nanowires growth, such as the Vapor–Liquid–Solid (VLS), which realized Si NWs with a too large size, thus preventing the quantum confinement effect, and therefore their luminescence at room temperature [68,69,70]. However, without other more complex procedures, it is very difficult to reach a small enough size to obtain quantum confinement [71]. Recently, we demonstrated the use of a modification of the Metal-Assisted Chemical Etching (MACE) approach to realize quantum-confined Si NWs—an ultra-thin gold (Au) layer used to obtain light-emitting Si NWs at RT with controlled morphology [72]. This is a low-cost process, maskless, and compatible with the industrial silicon technology. This process allows us to easily obtain very high Si NWs densities (10^12^ NW/cm^2^) in a very short time. Furthermore, it is possible to control the Si NWs average diameter by modifying the thickness of the deposited Au in order to obtain diameters small enough to have a quantum confinement emission at RT [57]. These structures have proved to be strategic platforms for the realization of highly selective sensors in detecting substances of different chemical nature and with extremely low limits of detection.

This paper will show the performance of a new gas detection platform based on Si NWs photoluminescence at RT. In particular, the Si NWs sensor will be realized without any type of decoration or surface functionalization for the detection of various gas targets such as acetone, ethanol, and ammonia exploiting both electrical and optical transduction. The versatility of this sensor will allow us to have a device capable of detecting variations in the chemical composition of the air even in the presence of a few ppm of pollutants.

## 2. Materials and Methods

### 2.1. Materials

Silicon wafers were purchased from Siegert Wafer (SIEGERT WAFER GmbH Charlottenburger Allee 7 52068 Aachen, Germany). Chemicals used for Si NWs realization as HF (48%), H_2_O_2_, and KI (gold etchant standard) were acquired from Sigma Aldrich (Merck KGaA Headquarters of the Merck Group Frankfurter Strasse 250 Darmstadt, 64293, Germany). Acetone, ethanol, and ammonia permeation tubes were procured from Fine Metrology srls (Via Vincenzo Monti 14, 98048, Spadafora, Messina, Italy).

### 2.2. Synthesis of Si NWs

**Figure 1:**
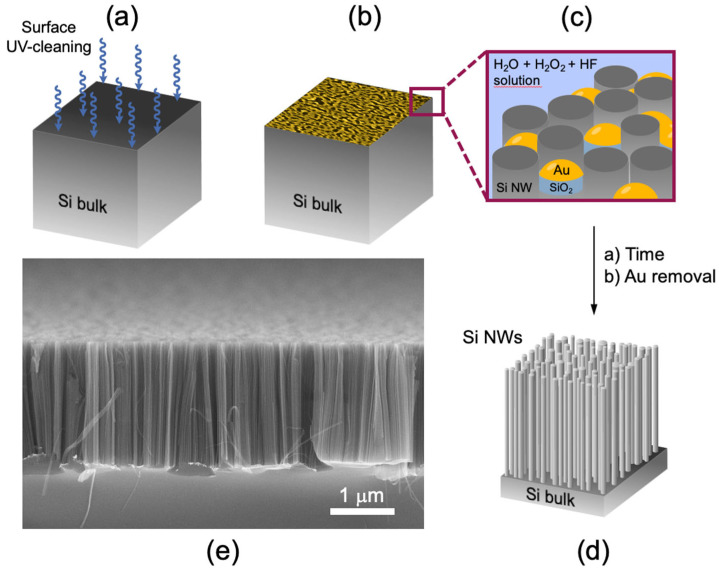
Scheme of the metal-assisted chemical-etching approach used for the Si NWs synthesis (**a**–**d**). SEM image of Si NWs in tilted cross-section view (**e**).

Silicon nanowires have been synthesized by using the MACE technique, which allows to obtain a high-density vertically aligned silicon nanowires array. Si NWs were realized from a (100)-oriented p++ commercial silicon wafer (~10^19^ Boron atoms/cm^3^) as the starting substrate. Firstly, Si wafers were oxidized by a UV ozone treatment (Figure 1a) for 2 min and then by immersing them in a 5% hydrofluoric acid (HF) aqueous solution for 5 min in order to remove the native silicon oxide from the surface. In this approach, an ultrathin percolative gold layer of 2 nm [72] was deposited in the SiO_2_-free silicon surface by electron beam evaporation at room temperature, as schematized in Figure 1b. This Au film is a noncontinuous layer, and the uncovered silicon regions had an average size of about 9 ± 2 nm [72]. The sample is then immersed in an aqueous solution of HF (5 M) and H_2_O_2_ (0.44 M) at RT. In these operating conditions, Au acts as a catalyst for the selective silicon oxidation under the gold interface. At the same time, HF reacted with the just-formed SiO_2_, dissolving it as H_2_SiF_2_ in solution. So, in the Au-covered silicon regions we can observe a selective etching of the Si, while in the gold-free silicon regions the formation of Si NWs happens, as pictured in Figure 1c. The Si NWs realized by using the MACE method have a length depending on the etching time and an average diameter of 7 ± 2 nm. These dimensions were obtained by the Energy-Filtered Transmission Electron Microscopy (EF-TEM) and Raman spectroscopy analysis [72]. All the steps of the synthesis are at RT, so the gold cannot diffuse inside the NW core, and it can be quickly removed by immersing the sample in a KI-based gold etching solution for 1 min in order to obtain an Au-free Si NWs array (Figure 1d). Figure 1 shows an image obtained by scanning electron microscopy (SEM) in a tilted cross section of the just-synthesized Si NW. As it is possible to observe, Si NWs have a length of ~2 µm and a huge calculated density equal to 10^12^ NWs/cm^2^. The Si NWs realized by this technique present the same crystalline orientation and doping of the starting Si substrate [73]. In addition, this approach guarantees a rapid Si NWs array realization through a low-cost and industrially compatible method. Moreover, this approach does not involve the use of masks and can be used on a large scale.

### 2.3. Structural, Optical and Electrical Characterization

Si NWs’ structural characterization was performed through a field emission Zeiss Sigma (Carl-Zeiss-Straße 22, 73447 Oberkochen, Germany) Scanning Electron Microscope (5 kV and an aperture of 30 μm).

A HR800 spectrometer (Horiba Jobin Yvon, HORIBA, Ltd. Head Office/Factory 2, Miyanohigashi, Kisshoin Minami-Ku Kyoto 601-8510, Japan) coupled to a CCD detector working at −62 °C was used to acquire the RT Photoluminescence spectra. The excitation wavelength is 476 nm obtained from an Ar^+^ laser, and the pump power onto the sample plan was in the 90–100 μW range. The spectra are all reported as normalized to take into account small differences about the laser pump power. Each spectrum we report in this paper is a statistical average obtained by carrying out different acquisitions on different points for each sample. The sample is placed in a closed cell equipped with a temperature controller in which the target gases are introduced. We have built a homemade setup capable of producing and transporting constant flows of known gas to the sample in order to record the sensor PL spectra in the presence of known gas concentrations.

A permeation tube system was used to drive a controlled amount of the various target gases used (acetone, ethanol and ammonia) inside the measuring system. The amount of gas that comes out from the tube membrane depends on the permeation rate through the tube membrane (which is a function of the chamber temperature), and on the gas carrier flow rate. The ppm concentration *c* of the gas target is calculated by Equation (1):

(1) $$c=\frac{v_{p}}{v_{\phi }}K$$

where v_p_ is the permeation rate expressed in ng/min, v**_ϕ_** is the flow rate expressed in cc/min, and K = 24.46/*M_W_* is a constant specific of the gas target used as a function of its molecular weight (*M_W_*). The permeation rate is a temperature-dependent parameter. So, all measurements reported in this article were performed by heating the permeation tube to 50 °C.

In the electrical measurements, the sensor response was measured by using a digital multimeter with a constant voltage of 2 V connected to a raspberry-pi in order to perform measurements even remotely. The sensor and the electrodes of the digital multimeter were interconnected through the realization of interdigitated electrodes. They were obtained by depositing 100 nm of Au on 5 nm of titanium (Ti) by electron beam evaporation through the use of specific aluminum masks. Gold was deposited subsequently with a titanium deposition in order to maintain the high vacuum (10^−6^ mbar), avoiding the oxidation of Ti.

## 3. Results and Discussions

Si NWs realized through the MACE technique were tested as a new detection platform for various hazardous gases. Above all, we tested our Si NWs sensor for the acetone, ethanol, and ammonia detection. All these gases are targets used for the development of even more sensitive and efficient forefront sensors for safety monitoring, not only in research and industrial laboratories, but also in urban environments (open and closed). The following paragraphs will show the optical and electrical performance of the silicon nanowires-based sensor with all these targets.

### 3.1. Optical Detection

**Figure 2:**
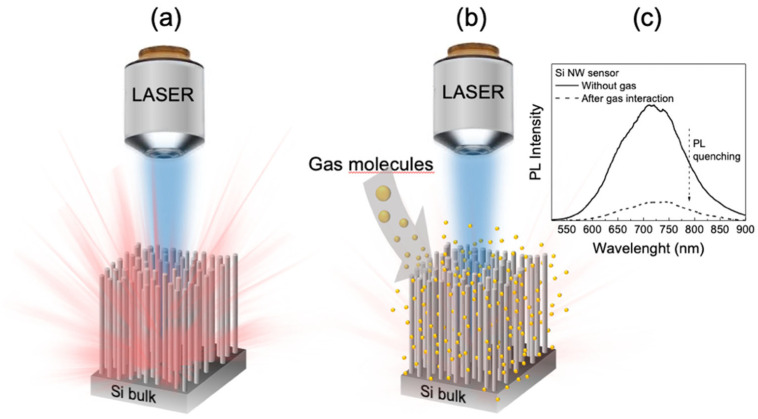
Schematic representation of the Si NWs PL variation before (**a**) and after (**b**) gas particles infiltration of inside the Si NWs matrix. (**c**) Visualization of the photoluminescence band quenching phenomenon following an interaction between Si NWs and a generic gas target.

Si NWs realized by using MACE approach are very efficient light-emitting systems even at RT. When Si NWs are excited with a light source, such as a laser radiation, an intense and stable visible/near-infrared light emission is observed, as schematized in Figure 2a. The emission band of the silicon nanowires is strongly influenced by the chemical neighbourhood and the refractive index of the medium inside the structure. It has already been reported that the interaction between Si NWs and target molecules of different natures causes a photoluminescence quenching (Figure 2b) as a function of the target concentration. In this case, when the gas target molecules are adsorbed on the surface of the nanowires, a decrease in the Si NWs PL intensity is observed (Figure 2c). The adsorption of the gas particles probably favors the population of new nonradiative levels, so the structure deactivates at the ground state losing its energy without photon emission. We will show the exploitation of the NWs PL quenching as a probe for the gas molecules detection.

**Figure 3:**
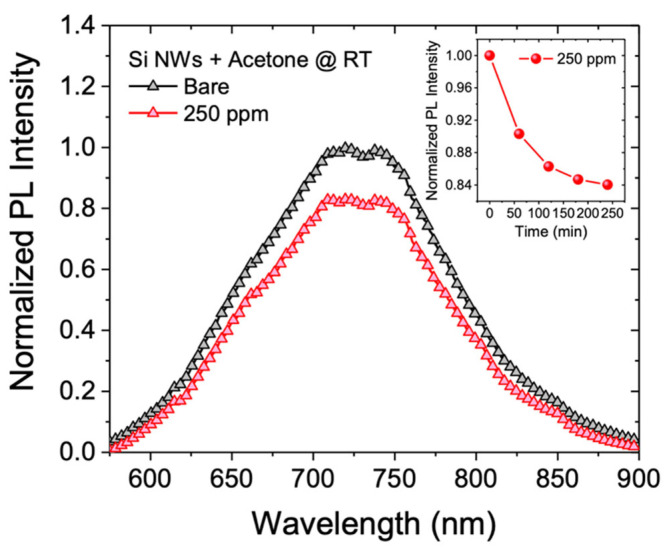
Normalized photoluminescent spectra at RT of the Si NWs sensor exposed to a 350 cc/min of N_2_ flow (black line) and 250 ppm of acetone (red line). The inset reports the sensor quenching kinetics.

The optical performance of our silicon nanowire-based sensor was tested by using two different gas targets: acetone and ethanol. Firstly, the sample was exposed to an overnight constant flow of 350 cc/min of N_2_ in order to obtain an oxygen-free chamber. Then, a mixture of N_2_ and acetone is introduced into the chamber and the photoluminescent spectra at room temperature are monitored over time (every hour), until no more PL intensity variations are observed. We tested the photoluminescence response of Si NWs in the presence of a controlled acetone concentration equal to 250 ppm by a permeation tube with a permeation rate v_p_ = 2966 ng/min. In Figure 3 the RT PL spectrum (black line) after stabilization in an inert atmosphere (N_2_) is shown; while the red line represents the nanowires PL recorded with 250 ppm acetone. All spectra show a wide luminescence band between 600 and 900 nm typical of quantum-confined Si NW.

Each spectrum shown in this paper is the result of numerous acquisitions recorded in different points of the sensor in order to obtain an accurate statistical mapping representative of the whole sample surface. To accurately estimate the change in the Si NWs PL intensity, the spectra were fitted by using a single Gaussian model. We calculated a photoluminescence intensity decreasing of the sensor by a factor of 1.2 with respect to the starting PL intensity. The inset in Figure 3 shows the normalized PL of the Si NWs sensor with 250 ppm of acetone as a function of time. The kinetic shows the typical saturation trend where a fast PL decreasing is observed in about 100 min, reaching 80% of the final value (obtained in the first two hours) and a second slower stretch, where sensor saturation is reached. These data show a clear response of the sensor even to small variations in the environmental chemical composition that can be used as a probe for the air quality monitoring.

**Figure 4:**
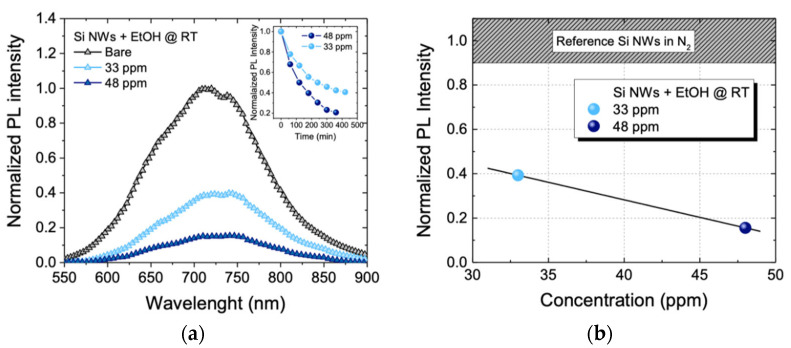
(**a**) Normalized luminescence spectra at RT of the sensor exposed to 350 cc/min of N_2_ (black line), 33 ppm of EtOH (cyan line), 48 ppm EtOH (blue line) and 180 ppm NO2 (blue line). (**b**) Sensor calibration curve at RT.

In order to verify the possibility of sensing other gaseous substances, we tested our sensor in the presence of small ethanol (EtOH) concentrations. We used two different concentrations of ethanol equal to 33 ppm and 48 ppm, both below the threshold limit value of 50 ppm for a driver security. In Figure 4a, the RT PL spectra of the sensor with the two different ethanol concentrations are reported. Also, in this case the sensor was exposed to an overnight 350 cc/min of N_2_ constant flow (black line). Then, the sensor was exposed to a constant flow of the N_2_/EtOH mixture at two different concentrations of EtOH. Next, 33 ppm (cyan line) and 48 ppm (blue line) of EtOH were blown by using a permeation tube with a permeation rate v_p_ = 457 ng/min. As can be observed, by increasing the EtOH concentration, the photoluminescence of the sensor decreases. Spectra were obtained by acquiring the PL of the nanowires every 30 min until no more variation in the emission intensity was observed. In the inset in Figure 4a, the sensor-normalized PL intensity as a function of time for the two EtOH concentrations are shown. All profiles show the same saturation trend as previously seen in the acetone sensing. It can be seen that sensor stabilization is achieved at lower PL values when the EtOH concentration is increased.

Also in this case, spectra were fitted by using the single Gaussian model and we calculated a signal decreasing by a factor of 2.5 for the EtOH concentration of 33 ppm (cyan spectrum) and by a factor of 6.4 increasing the concentration at 48 ppm (blue spectrum). The quenching of the luminescence of Si NWs-based sensors with an increasing target concentration has already been demonstrated [31,74].

The sensor calibration curve of the EtOH is reported in Figure 4b. The reference signal of the calibration curve, represented as the gray bar at the upper end of the graph, is relative to the Si NWs luminescence when exposed to the N_2_ flux only. The cyan dot is related to the sensor PL when exposed to 33 ppm of EtOH. This value decreases by about 60% with respect to the reference signal, while the blue dot refers to the sensor PL when it is exposed to 48 ppm of EtOH. This value decreases by about 84% with respect to the reference signal. With these results, we have clearly shown the sensitivity of the sensor both to different types of gas and even to a few ppm of acetone and ethanol. We can ascribe this behavior to the extremely high S/V ratio of these Si NWs, which allows strong interactions with these gases. The greater PL variations of the sensor in the presence of ethanol with respect to those observed in the presence of acetone demonstrate a greater sensor selectivity for ethanol. Furthermore, the great signal difference between the bare Si NWs and in presence of 33 ppm of ethanol suggests that the sensor may be able to detect much lower gas target concentrations. Commonly in literature, ethanol is detected in the range of a few ppm to over 100 ppm by using a high temperature of detection [75,76,77,78,79,80,81]. However, to the best of our knowledge, this is the first optical platform based on Si NWs with the RT luminescence detection. Then, low Limits of Detection (LODs) have been obtained by a low-cost Si-based platform working at RT, without any surface decoration or functionalization procedure. It is expected that the sensor LOD could be improved thanks to Si NWs surface functionalization with molecules able to selectively bind the various targets. This should also guarantee greater sensitivity more quickly.

### 3.2. Electrical Detection

**Figure 5:**
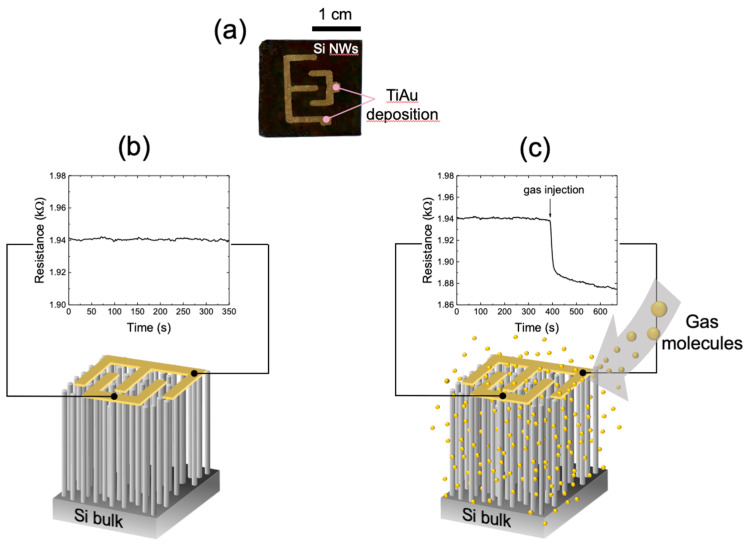
(**a**) Photograph of the sensor (2 × 2.3 cm^2^) composed of Si NWs platform (black color) covered by the interdigitated electrode. Pictures of the resistance variation of the silicon nanowires before (**b**) and after (**c**) the presence of the gas target. The electrodes are drawn in yellow above the Si NWs surface.

An Si NWs sensor made by the MACE technique was used for the sensing of various gaseous targets by exploiting the electrical transduction. For these measurements, the same type of substrate used for the optical measurements was tested. The image in Figure 5a reports a picture of the platform based on Si NWs where we deposited with an interdigitated geometry an Au layer of 100 nm onto 5 nm of Ti adhesion layer. The two electrodes onto the nanowire array were deposited by electron beam evaporation. They have a macro size with respect to the common microelectronic devices.

The realization of interdigitated electrodes above the platform is a crucial element as it allows to exploit the electrical conduction through the whole surface of the nanowires. This confines the influence of the surface interference [82], limiting the silicon bulk substrate contribution. We did not verify the absence of the bulk contribution that, however, according to the literature should be limited [83].

Figure 5b shows a scheme of our silicon nanowires-based sensor on which the interdigitated electrodes represented in yellow on their surface are depicted. The electrical conductivity of these silicon nanowires is extremely sensitive to even very small variations in the gas mixture chemical composition. Under controlled conditions, when there is no variation in the gas composition, the nanowires resistance remains constant over time. The measured resistance assumes a specific value based on both the morphological characteristics of the nanowires (length, diameter, doping) and the gas mixture composition inside the chamber. Figure 5b reports the sensor resistance stabilization after the insertion of a controlled N_2_ flow. As it is possible to observe, the resistance remains constant over time. When the gas mixture chemical composition of the chamber is changed, a very rapid variation in the sensor resistance is observed, as shown in Figure 5c. In this case, when a mixture of different gases (N_2_/NO_2_) is introduced into the chamber, a clear decrease in resistance is observed. We tested the electrical response of the sensor with two different gas targets, ammonia and acetone, and we measured the recovery times at different working temperatures.

To verify the response of the sensor to ammonia, several scans were performed by using controlled flows of NH_3_ at different working temperatures. Two measurements were performed at 70 °C, 100 °C, and 115 °C, in which the sensor resistance at a concentration of 9 ppm of NH_3_ of was measured.

Firstly, the sensor was exposed overnight to a constant flow of 350 cc/min of N_2_ in order to exclude the presence of adsorbed reactive oxygen species. Then, an exposure to a constant flow of 100 cc/min of a N_2_/NH_3_ gas mixture with an ammonia permeation rate of 623 ng/min ± 5% was carried out. This gas flow corresponds to 9 ppm of NH_3_ (our minimum tested concentration).

**Figure 6:**
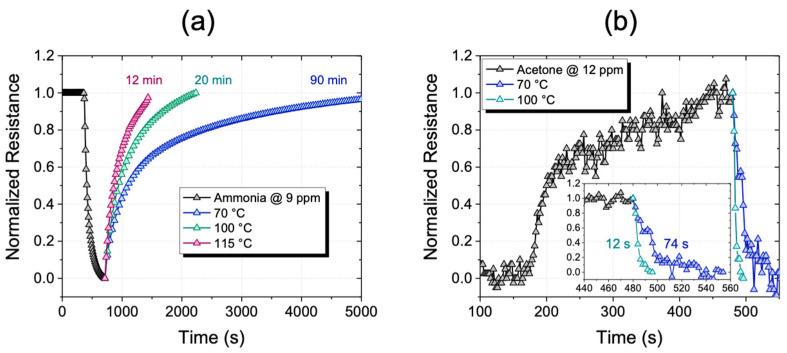
(**a**) Sensor electrical profile with 9 ppm of NH_3_ for 5 min. Recovery times at 70 °C, 100 °C, and 115 °C are shown with blue, green and red lines respectively. (**b**) Sensor electrical profile with 12 ppm of acetone for 5 min in the inset, an enlargement of the time scale on the sensor recovery time at temperatures of 70 °C (blue line) and 100 °C (green line).

In Figure 6a, the electrical profile of the sensor at the three working temperatures is shown. For the first 400 s, the curve is referred to the sensor stabilization under the overnight constant N_2_ flow of 350 cc/min. In these operating conditions, the sensor was stable with a constant resistance value. After that, 5 min long pulses of NH_3_ at 9 ppm were blown into the chamber. The gas pulse consists of a first down-hill section with a very steep slope related to the gas/sensor interaction. In the second part of the curve, the slope decreases over time due to the sensor surface saturation. Finally, in the third part of the curve, the electrical profile returns to its baseline showing that the sensor, in the presence of only N_2_, is subjected to desorption of gas molecules. The recovery time depends on the sensor temperature, as expected, since it refers to the physical–chemical interaction of the gas target onto the Si NWs surface. Indeed, a temperature-dependent variation in the recovery time was observed, as shown in Figure 6a. In order to show this effect, the pulse at 9 ppm of NH_3_ has been normalized for all the working temperatures of 70 °C, 100 °C, and 115 °C. As it can be possible to observe, an evident decreasing in the recovery time by increasing the working temperature occurs. The sensor recovery time at 70 °C is about 90 min and it is lowered to 20 min when the temperature is increased to 100 °C, while when increasing the temperature again to 115 °C it is still reduced to 12 min.

These perfectly aligned Si NWs have numerous air voids, which promote the diffusion of gas molecules. Therefore, by a slight increase in temperature, the desorption of the gas molecules from the active sites is favored. In this way, the molecules can be effectively removed from the sensitive layer through the channels between the nanowires. Recovery times can be considerably reduced by slightly increasing the working temperatures, thus obtaining an ease and fast sensor reuse.

The resistance variation is a function of the charge exchange between the adsorbed molecules and the Si NWs. It is well documented in the literature that the resistance variation depends on the doping of the sensor [84]. For doped n-type or undoped Si NWs, the resistance decreases under exposure to various concentrations of ammonia [85,86]. Ammonia is known to be a gas that has a reducing effect, whereby the molecules of NH_3_ when they adsorb on the surface of Si NWs transfer electrons to the crystalline nucleus of Si NWs according to Equation (2):

(2) $$NH_{3}\left(gas\right)\to NH_{3}^{+}\;\left(ads\right)+e^{-}$$

The adsorbed NH^3+^ ions raise the Fermi level of Si NWs at the top of the band gap, causing an increase in the sensor conductivity [87]. However, in the case of p-doped Si NW, the resistance is expected to increase as a result of electron injection [19,88,89]. Although the starting Si substrate used for the realization of NW has a p++ type doping, our results showed a decrease in the sensor resistance with ammonia vapors; this behavior, on the other hand, is common for n-type Si NW. However, an inversion in the conductivity is possible. This occurs when a highly defective oxide surface layer is present in the surface of the nanowires, leading to a high charge density [90]. Moreover, it is well documented in literature that, when the Si NWs diameter is much smaller than the space charge region width, the resistance change occurs [91]. As for our Si NWs, the extremely small diameters (less than 10 nm) are much lower than the layer for these nanostructures (greater than 100 nm), so an inversion of the resistance is very likely.

These measurements show huge potential in terms of electrical contacts scaling up, paving the way for the use of the silicon-based platform as gas microsensors.

To demonstrate the versatility of the sensor for also other types of gas target, the electrical profile of our Si NWs sensor with small acetone concentrations was also tested. To check the sensor performance to acetone, gas sensing measurements were performed in the presence of controlled flows of acetone at two working temperatures. Two measurements were performed at 70 °C and 100 °C. In these conditions, the sensor resistance at a concentration of 12 ppm of acetone was measured, as shown in Figure 6b. Firstly, we exposed the sensor to an overnight constant flow of 350 cc/min of N_2_. After the resistance stabilization, a controlled flow of 100 cc/min of a N_2_/Acetone gas mixture was blown for 5 min. Then, 12 ppm of Acetone (our minimum tested concentration) with a permeation rate v_p_ = 2966 ng/min ± 5% were inserted in the chamber. Also in this case, the sensor recovery time shows a temperature dependence. The pulse at 12 ppm of acetone with a S/N ratio of 10.3 was normalized for all the temperatures of 70 °C and 100 °C. A drastic decrease in the sensor recovery time by increasing the temperature is clearly visible. Sensor recovery times are considerably lower (in the order of seconds) than in the previous case of ammonia; this suggests a much faster gas exchange kinetics. Since the recovery times are extremely low, the insert in Figure 6b shows an enlargement of the temporal region relating to the sensor recovery at the two working temperatures in order to appreciate the variations. As can be possible to observe, the sensor shows a recovery time lower than 74 s at 70 °C, while the recovery time is lowered to less than 12 s when 100 °C is reached. The recovery time at 100 °C is already short enough, so a further temperature increase is of rare applicative interest.

In the literature, there are several examples of sensors for the detection of ammonia and acetone. The detection limits in the range of sub-ppm to over 200 ppm for ammonia [86,92,93,94,95,96,97,98] and between a few ppm and 100 ppm for acetone [99,100,101,102,103,104] have been reported. However, all these cited sensors work at high temperature, between 200 °C and 400 °C. Although the results obtained are not the best in terms of LODs, we were able to detect concentrations of a few ppm of ammonia and acetone by using working temperatures just above 100 °C. Despite the electrode macro size, the high signal/noise ratio calculated in all measurements guarantees a good starting point for a future optimization of our platform.

**Figure 7:**
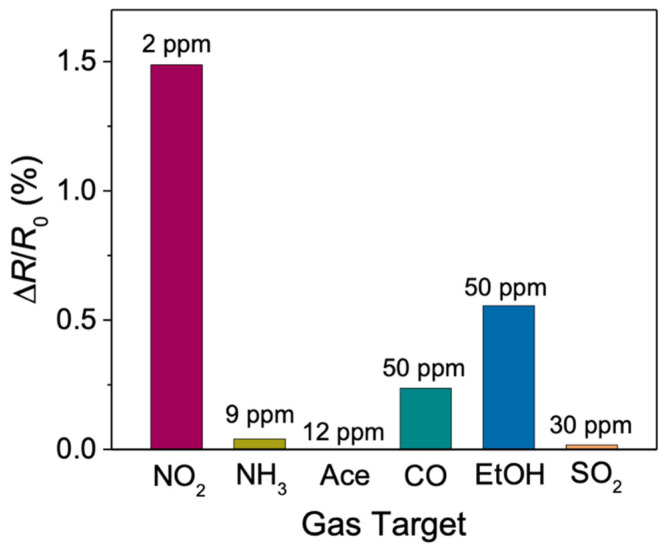
Histogram showing the percentage resistance variation of the Si NWs sensor for different gases tested.

We tested the electrical performance of our Si NWs sensor also with other gas targets in order to verify its selectivity, as shown by the histogram in Figure 7. All measurements were carried out at the working temperature of 70 °C in order to avoid variations due to thermal effects. The concentrations of the gases used correspond to the minimum amounts that can be supplied by our permeation tubes. The change in sensor resistance is shown after introducing 2 ppm of NO_2_, 9 ppm of NH_3_, 12 ppm of Acetone, 50 ppm of CO, 50 ppm of EtOH and 30 ppm of SO_2_. The change in electrical response, *S* (%), shown here is calculated by using Equation (3):

(3) $$S\left(\%\right)=\frac{\Delta R}{R_{0}}100=\frac{R_{0}-R}{R_{0}}100$$

where *R*_0_ is the resistance of the sensor (sensor baseline) and *R* is the resistance of the sensor exposed to the gas target (impulse resistance).

The resistance changes were calculated after gas pulses lasting 5 min for all measurements. As can be observed from the graph, the sensor response to 2 ppm of NO_2_ (a detailed discussion has already been described [74]) is significantly higher compared to the other gas target responses, even though their concentrations are much higher. The resistance variations in the presence of ammonia (0.04), acetone (3 × 10^−4^), and SO_2_ (0.017) are more than 20 times lower compared to those in the presence of NO_2_ (1.49). Despite these extremely low values, the sensor maintained a high S/N ratio enough for the clear detection of these gas targets.

## 4. Conclusions

A gas detection platform for acetone, ammonia and ethanol based on silicon nanowires has been reported. The interesting properties of MACE-grown Si NWs have been studied for the optical and electrical detection of low concentrations of extremely dangerous gases, mainly acetone, ethanol and ammonia. The sensor shows significant responses to all the tested gases both through photoluminescence and resistance variations, even below the threshold limits for human health and the environment. The possibility of using the same sensor to detect small concentrations of gas through both luminescence and electrical resistance variations makes it suitable and accessible for many users in different contexts. This is an aspect of extreme attention, especially in indoor workplaces where it is important to keep the quality of the air under control. Furthermore, based on the analysis of the PL and the variations in electrical resistance, it is possible that our platform may also be sensitive to lower gas concentrations than those shown here. Since the sample size used is relatively large, a decrease in recovery times can be observed only by decreasing the size of the interdigitate. However, small changes in temperature have been shown to reduce recovery times much more. Therefore, the temperature variation coupled to the sensor miniaturization can lead to a decrease in the recovery times. These results are an interesting springboard for the realization of next-generation gas sensing devices coupling a good limit of detection with an easy and compatible manufacturing flow.

## Data Availability

Data is contained within the article.
